# Transport cost to port though Brazilian federal roads network: Dataset for years 2000, 2005, 2010 and 2017

**DOI:** 10.1016/j.dib.2021.107070

**Published:** 2021-04-20

**Authors:** Daniel de Castro Victoria, Ramon Felipe Bicudo da Silva, James D.A. Millington, Valeri Katerinchuk, Mateus Batistella

**Affiliations:** a Embrapa Agricultural Informatics. Brazilian Agricultural Research Corporation (EMBRAPA). Avenida Andre Tosello 209 Campus da Unicamp Barão Geraldo. PO Box 6041 - 13083-886 Campinas, São Paulo, Brazil; b Center for Systems Integration and Sustainability, Department of Fisheries and Wildlife, Michigan State University. 115 Manly Miles Building 1405 S. Harrison Rd. East Lansing, MI 48823, United States; c Center for Environmental Studies and Research, State University of Campinas (NEPAM/UNICAMP), Rua dos Flamboyants, 155 - Cidade Universitária Campinas/SP - CEP: 13.083-867 Campinas, Brazil; d Department of Geography, King's College London, Strand, London WC2B 4BG, United Kingdom

**Keywords:** Infrastructure, Logistics, Exports, Transportation, Agricultural commodities, Soybean, GIS

## Abstract

Transport costs can play a significant role in agricultural exports and businesses profitability. It can also influence farmers’ decisions regarding cropland expansion, intensification or land abandonment. Thus, transport is useful to take into account when modeling and evaluating land use and cover change related to agriculture production. The dataset described in this article represents the *Infrastructure Capital* in the work presented by Millington et al. (2021) [1], in which the CRAFTY-Brazil model is used to evaluate the impacts of changing global demand for agricultural products on land use and cover change. This modeling required a transport cost dataset that spanned the model calibration period, was consistent through time and covered the entire study area. The most recent federal road network (for year 2017) obtained in vector format (shapefile) was joined to road section surface status tables for past years (2000, 2005 and 2010) in order to reconstruct the historic road network. Export ports handling agricultural commodities, and their years of operation, were identified. Both datasets were used to derive the relative transport cost to the nearest port for Brazil, for years 2000, 2005, 2010 and 2017.

## Specifications Table

**Table:** 

Subject	Transportation
Specific subject area	Transport cost of agricultural commodities based on road condition and distance to port
Type of data	Table Raster image (GeoTIFF) Vector network (GeoPackage and shapefile)
How data were acquired	All original data were downloaded from official governmental websites and processed using GRASS GIS v.7 [2].
Data format	Raw Processed
Parameters for data collection	Only data from official governmental sources were utilized. For some ports, the operational history was obtained from the port website or regional news reports.
Description of data collection	Federal road network shapefiles and road status data were downloaded from the Brazilian National Department of Transport Infrastructure (DNIT) website. The two datasets used were the National Road System (SNV – Sistema Nacional de Viação) for year 2017, obtained from the DNITGeo website [3] and the road status tables from the National Road Plan (PNV) for years 2000, 2005 and 2010, obtained from [4]. Information on export port operations were obtained from the Agrostat website [5]. Also, some news reports and information on the history of selected ports were consulted in order to establish the start of operations. All data were processed in GRASS GIS [2] and the commands and scripts used to recreate the final road network status for years 2000, 2005 and 2010, along with the transport cost maps, are included in this dataset.
Data source location	Institution: Embrapa Agricultural Informatics City/Town/Region: Campinas, SP Country: Brazil Primary data sources: DNITGeo Website Institution: National Department of Transport Infrastructure (DNIT) City/Town/Region: Brasilia, DF Country: Brazil URL: http://www.dnit.gov.br/planejamento-e-pesquisa/dnit-geo National Road Plan Institution: National Department of Transport Infrastructure (DNIT) City/Town/Region: Brasilia, DF Country: Brazil URL: http://www.dnit.gov.br/sistema-nacional-de-viacao/sistema-nacional-de-viacao Agrostat Institution: Ministry of Agriculture, Livestock and Food Supply City/Town/Region: Brasilia, DF Country: Brazil URL: http://indicadores.agricultura.gov.br/agrostat/index.htm
Data accessibility	Repository name: Mendeley Data
	Data identification number: DOI: 10.17632/6xbjzyz3th.2
	Direct URL to data: https://data.mendeley.com/datasets/6xbjzyz3th/2
Related research article	James D.A. Millington, Valeri Katerinchuk, Ramon Felipe Bicudo da Silva, Daniel de Castro Victoria, Mateus Batistella. Modelling drivers of Brazilian agricultural change in a telecoupled world. Environmental Modelling & Software. Volume 139, 2021, 105,024, ISSN 1364–8152.

## Value of the Data

• Transport cost and road infrastructure play an important role in agricultural exports. Information on how these costs change over time and across space can help understand the dynamics behind land use/cover change and the impact of infrastructure development on the agriculture sector. The dataset covers Brazil, a large and important producer of agricultural products traded internationally.
• The dataset can be used by researchers and decision-makers studying or concerned about land use/cover change and the impact of infrastructure on commodities export, how transport costs have changed over time, where new transportation and port infrastructure could be placed, or to identify bottlenecks in transport infrastructure. It could also be used to guide future infrastructure development plans.
• The dataset considers a relative cost difference among regions and road status and is accompanied by all codes and scripts used to create it. Thus, users of the dataset may adapt or improve upon the data, given their specific needs. All data were processed using open source software and are supplied in common formats supported by all major GIS. Thus, there is no need to acquire licenses to recreate or improve the results.
• Up-to-date roads network maps are available from the Brazilian transportation authority [3]. However, past road surface statuses are supplied as tables [4], not easily related to the road network, making it difficult to map past road conditions. The process described here links road sections to past surface status, resulting in transportation cost surfaces that are consistent and comparable across years.

## 1 Data Description

The dataset is organized in a folder structure that includes: i) a detailed description of the steps used to create the transport cost surface dataset; ii) the input datasets used to create the final transport cost iii) the actual transport cost dataset and iv) figures used in the detailed description report.

The contents of *root* folder of the dataset includes:

1. A brief description of the dataset folder structure (*readme.md* or *readme.txt)* in markdown format or text without formating marks, respectively);
2. a detailed description of the dataset creation process, including all GRASS GIS commands used, explanatory text and results obtained. This is presented in markdown format (*transport_cost.md*) and also in a rendered PDF file (*transport_cost.pdf*);
3. Additional code used in the data creation process including a bash script (*add_surface_code.sh*) that inserts road status codes to the road network vector layer used inside GRASS GIS and a python script (*fix_road_attrib.py*) that matches road sections in the road network vector layer to road surface status in the status table for each year. Files *matches_2000.txt, matches_2005.txt* and *matches_2010.txt* contain the matches obtained by the python script.

The *input* folder contains the original data used to create the transport cost dataset. It contains the road networks vector files, the road surface status tables and export port locations, as described bellow:

1. Folder *input/ports* contains the location of all Brazilian soy export ports (*SoyPorts.shp);* internal soy Ports (*internal_ports_soybean.shp*), that sends the commodity to an export port; and a table (*ports_year_active.ods* or *ports_year_active.xlsx*) detailing which ports were active in each year. This dataset was built using the Brazilian Agrostat plataform [5]. Both location datasets are in *shapefile* format, which can be opened by all major GIS software. The readme.md file describes the data sources;
2. Folder *input/roads/PNV* contains the Brazilian Federal Road network in *shapefile* format (*SNV_201710b.shp* and accompaning files). This represents the road network status in the year 2017 and was obtained from the DNIT - National Department of Transport Infrastructure [3]. A brief description of the dataset is given in *readme_SNV_roads.md*;
3. Folder *input/roads/Brazil_federal_roads_SNV* contains federal road section and surface status for years 2000, 2005 and 2010. It was obtained from the DNIT - National Roads Plan (PNV) [4] in Microsoft Excel format (ex.: *PNV2000.xlsx*) and converted to CSV (ex.: *PNV2000.csv)* to allow for data import into Grass GIS. CSVT files (ex.: *PNV2000.csvt)* indicates data field types, needed to import data in the correct format. A brief description of the dataset is given in *readme_road_network_status.md*;

The *output* folder contains the results of the data analysis conducted on the *input* data by following the steps detailed in *transport_cost.md*. It contains:

1. *road_network_ports_2000_2017.zip* - Vector layers (GeoPackage format) of export ports and reconstructed federal road network status for years 2000, 2005, 2010 and 2017 (Fig. 1). Data is supplied in Geopackage format that can be opened by all major GIS software. Due to its size, the GeoPackage file has been compacted;

**Fig. 1:**
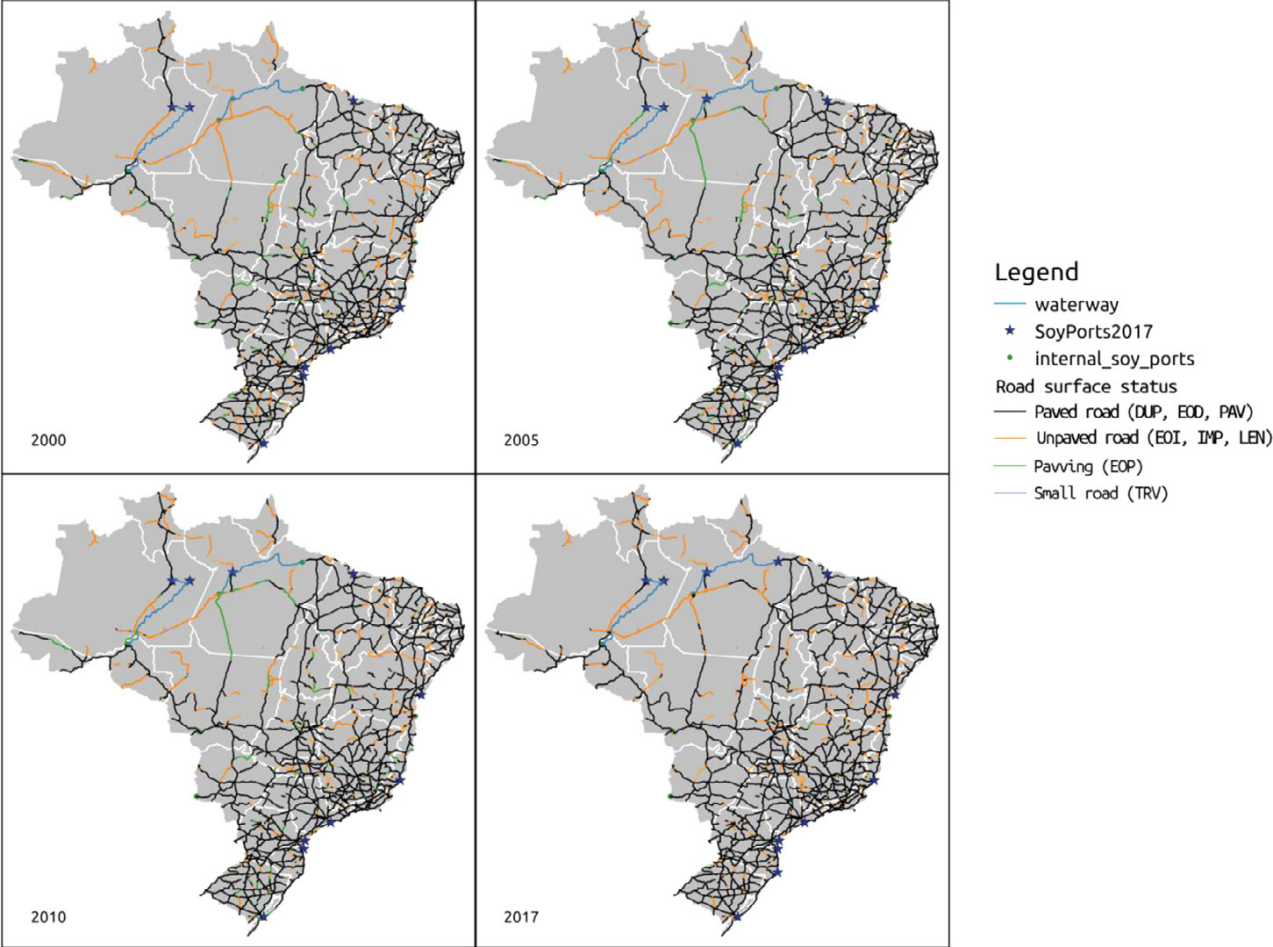
Reconstructed DNIT Road Network with simplified road surface status for years 2000, 2005, 2010 and 2017 and soy export ports active in each year, respectively. Three letters DNIT surface codes in parenteses.
2. *road_distanceYYYY.zip* - Raster images (GeoTIFF format) of relative cost distance to the nearest export port for the Brazilian territory, years 2000, 2005, 2010 and 2017 (Fig. 2). Dataset uses WGS84 Spatial Reference System (EPSG:4326) with 36 arcsec spatial resolution (approx 550 m). The GeoTiff format can be opened by all major GIS software.

**Fig. 2:**
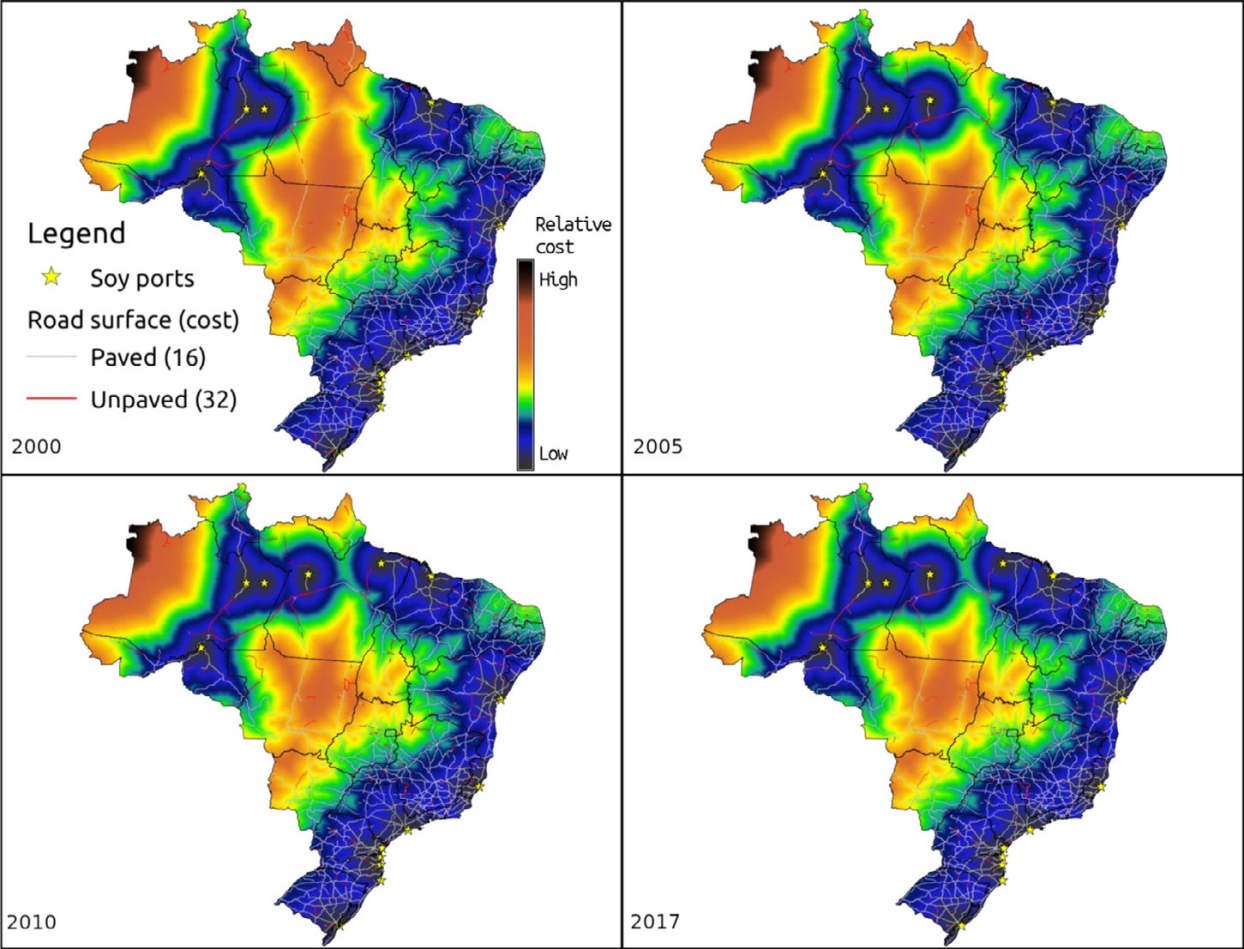
Cumulative cost distance to port, for years 2000, 2005, 2010 and 2017.

The *fig* folder contains figures used here and in the full detailed report (*transport_cost.md* or *transport_cost.pdf*) in PNG format. These include the reconstructed Brazilian federal road network with road surface status and port locations for years 2000, 2005, 2010 and 2017 and the relative cost distance to the closest export port for years 2000, 2005, 2010 and 2017.

A summary of the folder structure and its contents is given in Table 1.

**Table 1:** Folder structure, files contained in the dataset and detailed description.

Folder	Files	Description
figs	dnit2000_road_network_ports.pgw dnit2000_road_network_ports.png dnit2005_road_network_ports.pgw dnit2005_road_network_ports.png dnit2010_road_network_ports.pgw dnit2010_road_network_ports.png dnit2017_road_network_ports.pgw dnit2017_road_network_ports.png	Folder contains figures used in the full detailed report (transport_cost.md). Actual final dataset is in output folder. Brazilian federal road network with road surface status and port locations for years 2000, 2005, 2010 and 2017. Accompanying PGW files contains georeferencing information for the PNG files
	dnit_road_network_4years.png dnit_road_network_4years_no_ dannotations.png dnit_road_network_4years_no_ annotations_simple_legend.png	Figure with all four years of road network and ports, with, without annotations and simplified legend.
	dnit_road_distance2000.png dnit_road_distance2005.png dnit_road_distance2010.png dnit_road_distance2017.png	Relative cost distance to the closest export port for years 2000, 2005, 2010 and 2017
	dnit_road_distance_4years.png	Figure with all four years of road network cost distance to closest port.
input/ports	readme.md	Brief explanation of the ports input dataset
	internal_ports_soybean.cpg internal_ports_soybean.dbf internal_ports_soybean.prj internal_ports_soybean.sbn internal_ports_soybean.sbx internal_ports_soybean.shp internal_ports_soybean.shx	Shapefile with the location of internal and export ports that handle agricultural commodities. Shapefile format is comprised of several files, not all being mandatory
	SoyPorts.cpg SoyPorts.dbf SoyPorts.prj SoyPorts.shp SoyPorts.shx	Shapefile with location of export ports that handle agricultural commodities. Shapefile format is comprised of several files, not all being mandatory
	ports_year_active.ods ports_year_active.xlsx	Spreadsheet detailing amount of soy exported by each port, each year, in order to determine when each export port was active. Available in LibreOffice format (ODS) and Excel (XLSX)
input/roads/PNV	readme_road_network_status.md	Brief indication of data source and road status code and description in Portuguese and English
	PNV2000.csv PNV2000.csvt PNV2000.xlsx PNV2005.csv PNV2005.csvt PNV2005.xlsx PNV2010.csv PNV2010.csvt PNV2010.xlsx	Tables indicating federal road section and surface status for years 2000, 2005 and 2010. Original data from DNIT is in XLSX format. Converted to CSV for data import into Grass GIS. CSVT file indicates data field types, needed to import data in the correct format
input/roads/Brazil_ federal_roads_SNV	readme_SNV_roads.md	Brief indication of data source
	SNV_201710B.cpg SNV_201710B.dbf SNV_201710B.prj SNV_201710B.sbn SNV_201710B.sbx SNV_201710B.shp SNV_201710B.shx	Shapefile with the federal road network for the year 2017. Contains road surface status
output	road_distance2000.zip road_distance2005.zip road_distance2010.zip road_distance2017.zip	Raster images (GeoTIFF format) of relative cost distance to the nearest export port for the Brazilian territory, years 2000, 2005, 2010 and 2017. Dataset uses WGS84 Spatial Reference System (EPSG:4326) with 36 arcsec spatial resolution (aprox 550 m)
	road_network_ports_2000_2017.zip	Vector layers (GeoPackage format) of export ports and reconstructed federal road network status for years 2000, 2005, 2010 and 2017.
. (root)	readme.md and readme.txt	Brief directory structure description
	add_surface_code.sh	Bash script to insert road surface status code to 2017 road network vector layer
	fix_road_attrib.py	Python script to identify matches from road sections in the road network vector layer to road surface status table for each year.
	matches_2000.txt matches_2005.txt matches_2010.txt	Results from the road section and status match python script.
	transport_cost.md transport_cost.pdf	Detailed description of dataset creation, including GRASS GIS commands used and results obtained. Report is in Markdown format (md) and is accompanied by a rendered PDF.

## 2 Experimental Design, Materials and Methods

This dataset was constructed to be used as the *Infrastructure Capital* input for the CRAFTY-Brazil model [1].

### 2.1 Road network reconstruction

A detailed federal road network vector dataset was downloaded from the National Department of Transport Infrastructure (DNIT) geospatial database [3]. This database contains road network vector layers, in shapefile format, beginning in 2013. The dataset for 2017 was used, along with road number, the road section code, road section start and stop km marks and road surface status, with the following classification (original class code and portuguese description in parentheses):

1. Planned (“PLA - Planejada”)
2. Unpaved network (“Rede não pavimentada”)
  1. Natural terrain. Open road, not conforming to construction regulations (“LEN - leito natural”)
  2. Under construction to be implemented (“EOI - em obras implantde implantação”)
  3. Established unpaved road (“IMP - implantada”)
  4. Under construction. Established road being paved (“EOP - em obras pavimentação”)
3. Paved network (“rede pavimentada”)
  1. Paved road single lane (“PAV - pista simples”)
  2. Second lane under construction. Paved road being duplicated - (“EOD - em obra de duplicação”)
  3. Double lane (“DUP - pista dupla”)

In order to reconstruct road networks and surface status prior to year 2013, road section information for the years 2000, 2005 and 2010 were obtained from the National Road System (SNV) [4]. This information comes as tables known as National Roads Plan (PNV – Plano Nacional de Viação) and includes information on road number, road section identification code, the Brazilian state crossed by the road, section start and stop landmarks and kilometer marks, section length and road surface status.

Unfortunately, road section identification codes from the vector network dataset for 2017 does not match all section codes found in the 2000, 2005 and 2010 tables. After joining the vector network dataset with the surface status tables, some road segments will remain unclassified. Thus, the best possible match between the vector network sections and surface status tables were obtained based on road number, Brazilian state and section start and stop kilometer marks. A python script (*fix_road_attribute.py*) was used to automate this process and identify possible matches. The script identifies all sections from the road network dataset without surface type information. It then searches the status table for sections with the same road number, that crosses the same Brazilian state and is either entirely within the section identified in the road network dataset or covers its start or end portions. Since this procedure can return more than one match, the most frequent surface type is assigned to the road section.

### 2.2 Port activity dataset

Brazilian ports that export agricultural commodities were identified based on information from Agrostat [5], a database for Brazilian agricultural exports, which includes among other things, the ports involved in the transaction. This information, along with regional reports from selected ports, were used to derive an indication of when each port started operations (Table 2). Ports with very low agricultural exports were removed from the dataset and the final surface cost analysis.

**Table 2:** Brazilian ports with agricultural commodities exports in 2000, 2005, 2010 and 2017 (0 indicates no exports. 1 indicates exports).

Port	2017	2010	2005	2000	Observations
Manaus	1	1	1	1	
Itacoatiara*	1	1	1	1	
São Luis	1	1	1	1	
Aracajú	1	0	0	0	Soy exports only in 2017. But is REMOVED from the analysis since volume is very low (< 0.5% exports)
Salvador	1	1	1	1	No soy export from 2002 to 2005 but active in other commodities
Ilheus	1	1	1	1	REMOVED: < 0.5% soy exports
Vitória	1	1	1	1	
Barcarena	1	1	0	0	Focused on mineral exports. Started operating ag. commodities in 2007 (cocoa) and 2008 (meat)
Belém	0	0	0	0	
Santarém	1	1	1	0	
Santos	1	1	1	1	
Paranaguá	1	1	1	1	
São Francisco do Sul	1	1	1	1	
Itajaí	1	1	1	1	Large meat export despite low soy exports
Imbituba	1	1	1	1	Old port that started exporting meat in year 2000
Rio Grande	1	1	1	1	
Porto Murtinho	1	0	1	0	REMOVED: < 1% exports, varies in time
Cáceres	0	0	1	1	REMOVED: < 1% exports, varies in time - not operational anymore
Porto Velho⁎⁎	1	1	1	1	Internal port, treated as export port

⁎ AgroStat export data from 2007 to 2017 is 0 (zero) for this port. But data seems to be mixed with the Manaus port. Since the ports are very close, both ports were assumed open all the time.

⁎⁎ Porto Velho (RO) is a transbordo port (internal), which sends soy to Santarém and then exports. It's included in a separate dataset, which is appended to the export ports and treated as such. Rondonia Port and Waterways web portal contains a report stating that in 2004 1.5 million tons of soy were exported, thus the port was active at least in 2004. ANTAQ: Port exported 1 million tons of soy in 2002 (http://web.antaq.gov.br/Portal/Anuarios/Portuario2002/InformacoesGeraisPortos/Portos/PortoVelho.htm).

### 2.3 Transport cost analysis

Relative transport costs in Brazil were estimated as the cumulative cost of moving from each grid cell to the closest export port. Cost was considered as a unitless indicator of the difficulty in crossing a cell, as a function of surface type (Table 3). Its values were defined considering the differences between each surface type and not the actual travel cost. Thus, results indicate a relative cost difference from different regions and years for the Brazilian territory.

**Table 3:** Cost to traverse a grid cell based on road surface type.

Surface type	Cost
No road network	50
Unpaved road	36
Paved road	16

### 2.4 Data processing

All data were processed using the open source GRASS GIS software [2]. Manipulation of vector data attribute tables were done using GRASS GIS database capabilities [6], especially it's ability to use SQL (Structured Query Language) and perform direct connection to the underlying Relational Database Management System (RDBMS). Cost surface was calculated using GRASS GIS *r.cost* command. A detailed description of the algorithm can be found in *r.cost* man page [7]. A detailed explanation of all commands used, sequences and expected results are given in the markdown file *transport_cost.md* (also available in PDF form). An overview of the process carried out in GRASS GIS is given bellow. For a better comprehension of GRASS GIS capabilities and concepts, refer to the Grass wiki, specially GRASS Help [8] and GIS Concepts in GRASS [9].

1. All process was carried out using EPSG:4326 projection;
2. Vector maps (road networks and pots locations) where imported into the GRASS database using *v.in.ogr* and command;
3. Ports layer were separated into different layers containing only the active ports for each year;
4. All planned roads from the 2017 road network layer where removed;
5. The 2010 road network layer was created by copying the 2017. The road surface status was removed and populated by joining with the 2010 road surface status table, based on road section id. Section ids not present in the 2010 road surface status table were joined based on road number and start/end km marks, using the *fix_road_attribute.py* code;
6. Step 5 was repeated for years 2005 and 2000;
7. For all years, cost value was added to the road network layer and convert to raster format. Active ports dataset was also converted to raster format;
8. Cost analysis (*r.cost* command) was carried out for all years, using the ports layer as the starting point

## Funding

This work was supported by Embrapa and the São Paulo Research Foundation (FAPESP), processes 14/50628–9, 15/25892–7, and 18/08200–2. We gratefully acknowledge the funding support by the UK Natural Environment Research Council, under grant reference NE/M021335/1.

## Declaration of Competing Interest

The authors declare that they have no known competing financial interests or personal relationships which have, or could be perceived to have, influenced the work reported in this article.

## Appendix Supplementary materials

Image, application 1
