# Supplementary material for: Transport cost to port though Brazilian federal roads network: Dataset for years 2000, 2005, 2010 and 2017
Source: Data Brief. 2021 Apr 20;36:107070. doi: 10.1016/j.dib.2021.107070 (PMC8131979; doi:10.1016/j.dib.2021.107070)
Supplement: Supplementary file 1 [file mmc1.pdf]

Transport Cost to Ports through Brazilian Federal Roads Networks: Dataset for years 2000, 2005, 2010 and 2017

Authors: Daniel de Castro Victoria, Ramon Felipe Bicudo da Silva, James D. A. Millington, Valeri Katerinchuk, Mateus Batistella
Email: daniel.victoria@embrapa.br

Document revision

Date	Description	Author
2017-12-27	Initial Version. Merry Xmas	DCV
2018-01-18	Change road network source to DNIT	DCV
2018-01-18	Remove unused or low traffic ports	DCV
2020-04-16	Fix ports on/off table (esp. Salvador, Barcarena and Imbituba)	DCV
2021-03-30	Follow DIB guidelines and update references	DCV

These are the steps taken to create the transport cost maps for the Belmont Project.
All processing is done on GRASS GIS.

This text is a companion to the data article:
D. D. C. Victoria, R. F. B. da Silva, J. D. A. Millington, V. Katerinchuk and M. Batistella, "Transport Cost to Ports through Brazilian Federal Roads Networks: Dataset for years 2000, 2005, 2010 and 2017". Data in Brief, *in press*

This dataset was produced for use in:
James D.A. Millington, Valeri Katerinchuk, Ramon Felipe Bicudo da Silva, Daniel de Castro Victoria, Mateus Batistella. Modelling drivers of Brazilian agricultural change in a telecoupled world. Environmental Modelling & Software. Volume 139, 2021, 105024, ISSN 1364-8152. https://doi.org/10.1016/j.envsoft.2021.105024.

TL/DR

DNIT supplies the SNV (Sistema Nacional de Viação) shapefile, which contains the road network for the year 2017, divided by road sections (with section ID, start and end KM marks) and a road surface status. DNIT also supplies the PNV (Plano Nacional de Viação) for different years (2000 to 2017). This is a table that contains road sections (with section ID, start and end KM marks) and surface status. We used the codes in the 2017 map, along with start-end road kilometer marks, with the DNIT status table for past years in order to derive road surface status for years 2000, 2005, 2010 and 2017.

PNV web site: http://www.dnit.gov.br/sistema-nacional-de-viacao/sistema-nacional-de-viacao
SNV web site: http://www.dnit.gov.br/planejamento-e-pesquisa/dnit-geo

Another possible dataset is the *Milton Santos Atlas* with road and rail networks for different years. But we choose to use the DNIT + PNV since its contains a more detailed road network.

No rail or internal waterways: For now we are not considering rail network in the cost maps, since it is a bit more complicated to model specific points in the network that the commodities can get in/out (railway stations). Thus, NO RAIL in this analysis. Also, internal waterways transports are modeled indirectly, considering that regional ports (such as Porto Velho - RO) acts as export ports. A more detailed modeling would have to treat waterways similar to railways, with regional ports as stations.

Ports activities: Data from agricultural commodities exports for each port (mainly soy but other commodities were considered, such as corn, meat and sugarcane), were used to identify when each port was active. Exports data were obtained from Agrostat.

We also used local and national news articles in order to study the port history and better identify the starting year of port activity. See bellow for a more detailed description.

Datasets

Road network from DNIT and status table

DNIT road network (SNV_201710B) is in shapefile format and contains roads segments for the year 2017. The attribute table contains (among other columns):

1. vl_br: Road number
2. sg_uf: State
3. vl_codigo: unique code for the road segment (which is not consistent across years)
4. vl_km_inic: starting km of the road segment
5. vl_km_fina: ending km
6. leg_multim: road surface status (this is probably a summary of several other columns). Long text
7. *vl_surface: this column was created based on leg_multim (using 3 letter code)*

The PNV tables (Plano Nacional de Viação) describe the road network status at different years (2000, 2005 and 2010) and contains (among other columns):

1. br: road number
2. uf: state
3. codigo: road segment code (not consistent across years)
4. km_ini: starting km
5. km_fim: ending km
6. superficie: road surface status (3 letter code)

The road surface status (and its 3 letter code) used by both SNV and PNV are:

1. Planejada (PLA) - *planned*
2. Rede não pavimentada - *unpaved network*
    i. leito natural (LEN) - *Natural terrain - just an opened road, not conforming to construction regulations*
    ii. em obras de implantação (EOI) - *under construction to be implemented*
    iii. implantada (IMP) - *implemented, unpaved road*
    iv. em obras pavimentação (EOP) - *under construction. IMP road being paved*
3. rede pavimentada - *paved network*
    i. pista simples (PAV) - *paved road*
    ii. em obra de duplicacao (EOD) - *second lane under construction. PAV road being duplicated*
    iii. pista dupla (DUP) - *Duble road*

A description of each surface status can be found in http://www.dnit.gov.br/download/rodovias/rodovias-federais/terminologias-rodoviarias/terminologias-rodoviarias-versao-11.1.pdf

Agriculture commodities Export Ports

A dataset containing agricultural commodities export ports and the year each started operation was created. We considered soybean export volume at each year as a proxy to determine when a port was actively exporting agricultural commodities. But export volumes of other agricultural commodities were also evaluated, especially corn, meat and sugarcane. This was based mainly on data gathered from Agrostat, an agriculture import/export database from the Ministry of Agriculture (MAPA). Ports that represented only a small fraction of total soy exports were excluded. The table bellow lists the port and the periods of activity.

Active soy export ports per year:

active in	2017	2010	2005	2000	Obs

active in	2017	2010	2005	2000	Obs
MANAUS	1	1	1	1	
ITACOATIARA*	1	1	1	1	
SAO LUIS	1	1	1	1	
ARACAJU	1	0	0	0	Soy exports only in 2017. But is REMOVED from the analysis since volume is very low (< 0.5% exports)
SALVADOR	1	1	1	1	No soy export from 2002 to 2005 but active in other commodities
ILHEUS	1	1	1	1	REMOVED: < 0.5% soy exports
VITORIA	1	1	1	1	
BARCARENA	1	1	0	0	Focused on mineral exports. Started operating ag. commodities in 2007 (cocoa) and 2008 (meat)
BELEM	0	0	0	0	REMOVED: no soy exports
SANTAREM	1	1	1	0	
SANTOS	1	1	1	1	
PARANAGUA	1	1	1	1	
SAO FRANCISCO DO SUL	1	1	1	1	
ITAJAI	1	1	1	1	Large meet export despite low soy exports
IMBITUBA	1	1	1	1	Old port that started exporting meat in year 2000
RIO GRANDE	1	1	1	1	
PORTO MURTINHO	1	0	1	0	REMOVED: < 1% exports, varies in time
CACERES	0	0	1	1	REMOVED: < 1% exports, varies in time - not operational anymore
PORTO VELHO**	1	1	1	1	Internal port, treated as export port

* AgroStat export data from 2007 to 2017 is 0 (zero) for this port. But we believe the data is mixed with the Manaus port. Since both are very close by, we choose to keep both ports on all the time.

** Porto Velho (RO) is a *transbordo* port (internal), which sends soy to Santarém and then exports. It's included in a separate dataset, which is appended to the export ports and treated as such.

Web portal of the Rondonia port and waterways has a report saying that in 2004 the port exported 1.5 million tons of soy thus the port was active at least in 2004.
Website: http://www.rondonia.ro.gov.br/soph/institucional/relatorio-anual-de-atividades/
Wikipedia: Port of Porto Velho is from 1997. It's administrated by SOPH.

ANTAQ: Port exported 1 million tons of soy in 2002 (http://web.antaq.gov.br/Portal/Anuarios/Portuario2002/InformacoesGeraisPortos/Portos/PortoVelho.htm).

Cost analysis

Transport cost in Brazil are estimated as the cumulative cost of moving from each grid cell to the closest export port. Cost to traverse each grid cell is related to road presence and surface type, as follows:

Cost for traversing a grid cell by road surface type:

Type	Cost
no network	50
Unpaved road	36
Paved road	16

For this analysis we treated the cost as an unitless indicator of the difficulty in crossing a cell. Thus, results will give a relative cost difference between regions and years in Brazil. A detailed description of the cost algorithm can be found in r.cost man page.

Grass processing

Here we detailed the steps taken in GRASS GIS in order to conduct the analysis.

All work is done in Grass.

- Location: brasil_wgs84
- mapset: logistica

Prepare ports vector maps

```
v.in.ogr -o input=inputs/ports/SoyPorts.shp output=SoyPorts
```

Removing ports that don't have significant exports.

```
v.edit map=SoyPorts tool=delete where="NAME='CACERES'"
v.edit map=SoyPorts tool=delete where="NAME='PORTO MURTINHO'"
v.edit map=SoyPorts tool=delete where="NAME='ARACAJU'"
v.edit map=SoyPorts tool=delete where="NAME='ILHEUS'"
v.edit map=SoyPorts tool=delete where="NAME='BELEM'"
```

```
v.extract input=SoyPorts output=SoyPorts2000 where=active00=1 --overwrite
v.extract input=SoyPorts output=SoyPorts2005 where=active05=1 --overwrite
v.extract input=SoyPorts output=SoyPorts2010 where=active10=1 --overwrite
v.extract input=SoyPorts output=SoyPorts2017 where=active17=1 --overwrite
```

Internal soy ports. Only interested in Porto Velho port, in operation at least since 2000. Other ports are already included in SoyPorts Vector.

```
v.in.ogr input="inputs/ports" layer="internal_ports_soybean" output="internal_soy_ports"
```

Prepare road network for different periods

Import table and shapefiles into GRASS GIS

```
v.in.ogr --overwrite input="input/roads/Brazil_federal_roads_SNV/SNV_201710B.shp" layer="SNV_201710B"
output="SNV_201710B" min_area=0.0001 type="" snap=1e-13
```

Cleaning SNV roads dataset for the year 2017 network

For year 2017, all roads that are still planned in 2017 will be removed. We are keeping the information regarding paved and unpaved roads.

```
v.extract input=SNV_201710B output=dnit2017 where="leg_multim not like 'Planejada%'"
v.db.addcolumn dnit2017 columns='vl_surface TEXT'
```

Use `add_surface_code.sh` to update the `vl_surface` column.

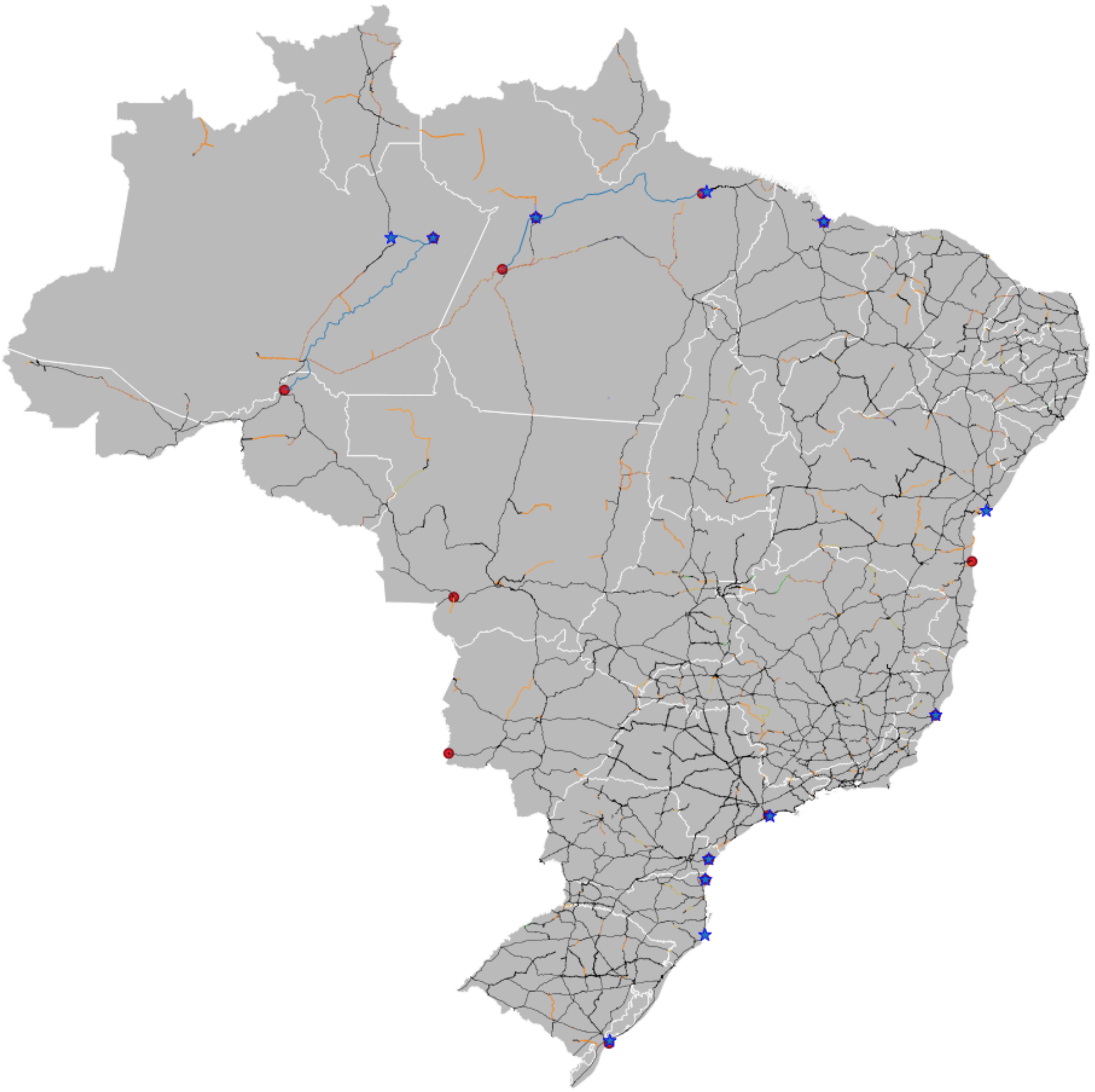

DNIT road network and soy export ports (blue stars) for year 2017. Red dots are internal soy ports, that transfer to export ports through waterways (blue lines). Paved roads in black. Dirt roads in orange.

Reconstructing 2010 road network

We will start by using the 2017 road network and finding which `vl_codigo` in the map exists in the PNV2010 table, `codigo` column. However, since the road segment code **is not consistent across years**, for road segments that we could not find a corresponding code in the PNV2000 table, we will then search based on road number, state and start/end km.

```
g.copy vect=dnit2017,dnit2010
```

Import PNV table. The CSV file is obtained from the XLS files downloaded from DNIT website. Don't forget to include column type description, using *.csvt* file with the same name as the CSV file.

For the year 2010, some road segments had a coincident 'state road' with a diverging road surface status. I'm considering the 'state road' status, when present.

```
db.in.ogr input=input/roads/PNV/PNV2010.csv output=pnv2010
```

Now, join the pnv2010 data to the dnit2010 map.

```
v.db.join map=dnit2010 column=vl_codigo other_table=pnv2010 other_column=codigo
```

How many of each road type there are?

```
db.select sql='select superficie, count() from dnit2010 group by superficie'
```

superficie	count()
	1000
DUP	842
EOD	80
EOI	4
EOP	145
IMP	356
LEN	116
PAV	3899
PLA	86
TRV	28

There are 86 planned road segments and 1000 that could not be found in the pnv2010 table. We start by finding some information for the segments with no road surface status. Then we remove the planned segments.

In order to find some road surface status information, a python code was written (`fix_road_attrib.py`) that uses the road number, state and start/end kilometer to find possible matches. We then attribute the most common surface status of the past road conditions to the vector map.

```
./fix_road_attrib.py dnit2010 pnv2010 -r matches_2010.txt
```

On the above command you need to use the flag *-u* to actually alter the table

Of the 1002 road segments with no surface status, the codes will be converted to:

new status	count
PAV	585
EOD	25
EOI	3
LEN	23
IMP	69
None	27
EOP	48
PLA	83
DUP	121

new status	count
TRV	16

After the matching routine, the distribution of road segment status are:

superficie	count()
	27
DUP	963
EOD	105
EOI	7
EOP	193
IMP	425
LEN	139
PAV	4484
PLA	169
TRV	44

Removing road sections with no surface information or planed status.
obs v.edit tool=delete does not alter the attribute table. So it's best to use v.extract for this job

```
v.extract -r input=dnit2010 where="superficie is 'PLA' or superficie is null" output=dnit2010c
g.rename --o vect=dnit2010c,dnit2010
```

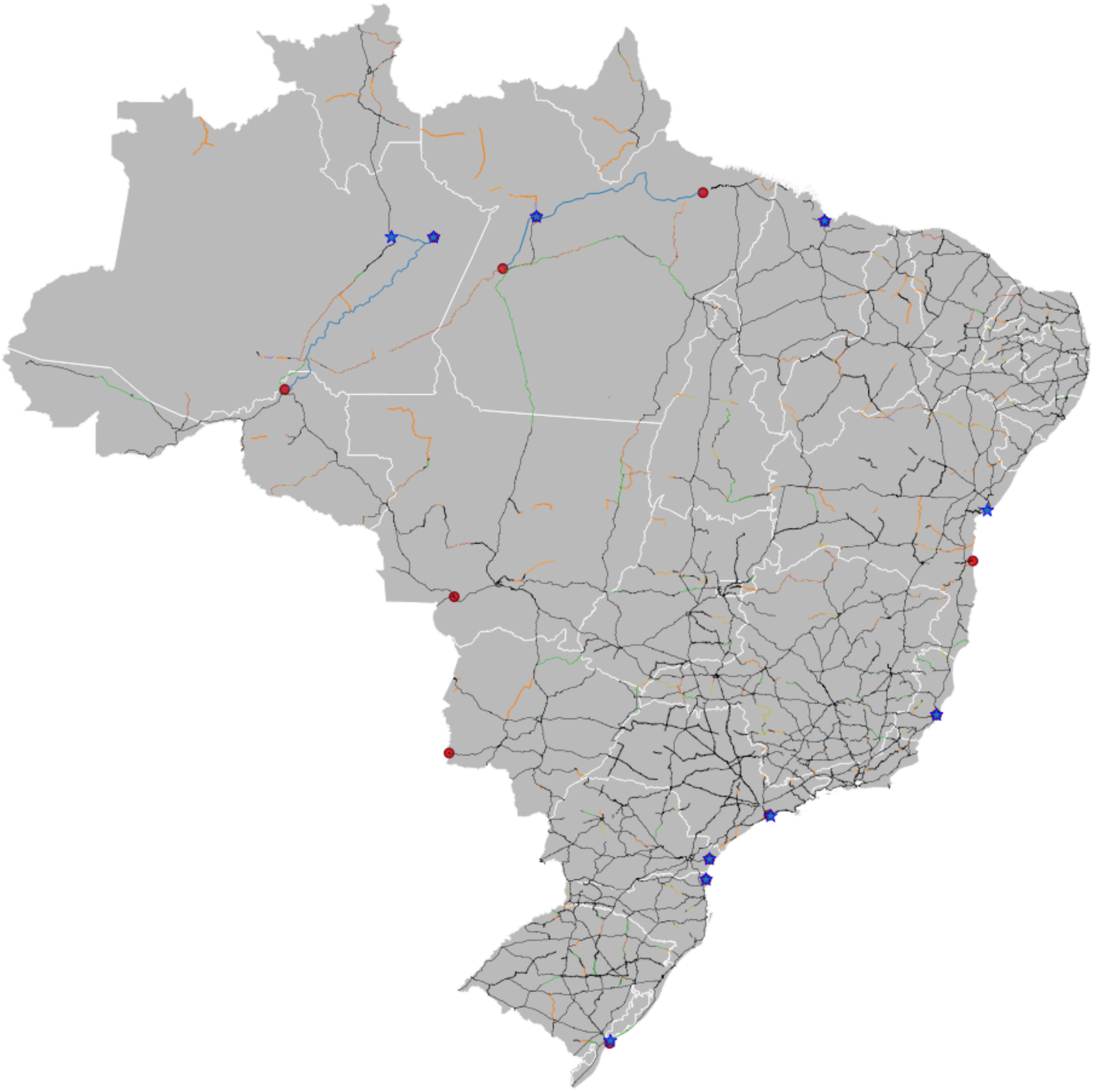

DNIT road network and soy export ports (blue stars) for year 2010. Red dots are internal soy ports, that transfer to export ports through waterways (blue lines). Paved roads in black. Dirt roads in orange.

Reconstructing 2005 road network

We start from the 2010 road network but must first remove the columns in the map that were inserted by the join

```
g.copy vect=dnit2010,dnit2005
v.db.dropcolumn map=dnit2005
columns=br,uf,codigo,local_inicio,local_fim,km_inic,km_fina,extensao,superficie_federal,estadual_coincidente,superficie_estadual,superficie
db.in.ogr input=input/roads/PNV/PNV2005.csv output=pnv2005
v.db.join map=dnit2005 column=vl_codigo other_table=pnv2005 other_column=codigo
```

How many of each road type there are?

```
db.select sql='select superficie, count() from dnit2005 group by superficie'
```

superficie	count()
	1171
DUP	645
EOD	40
EOI	8

superficie	count()
EOP	165
IMP	361
LEN	120
PAV	3782
PLA	40
TRV	28

Finding the status attribute for 1290 road sections.
`./fix_road_attrib.py dnit2005 pnv2005 -r matches_2005.txt`

New status of the unclassified roads will be:

Status	count
PAV	752
EOD	14
EOI	7
LEN	37
IMP	100
None	44
EOP	59
PLA	48
DUP	107
TRV	3

After substitution, road status are:

superficie	count()
	44
DUP	752
EOD	54
EOI	15
EOP	224
IMP	461
LEN	157
PAV	4534

superficie	count()
PLA	88
TRV	31

Removing road sections with no surface information or planed status

```
v.extract -r input=dnit2005 where="superficie is 'PLA' or superficie is null" output=dnit2005c
g.rename --o vect=dnit2005c,dnit2005
```

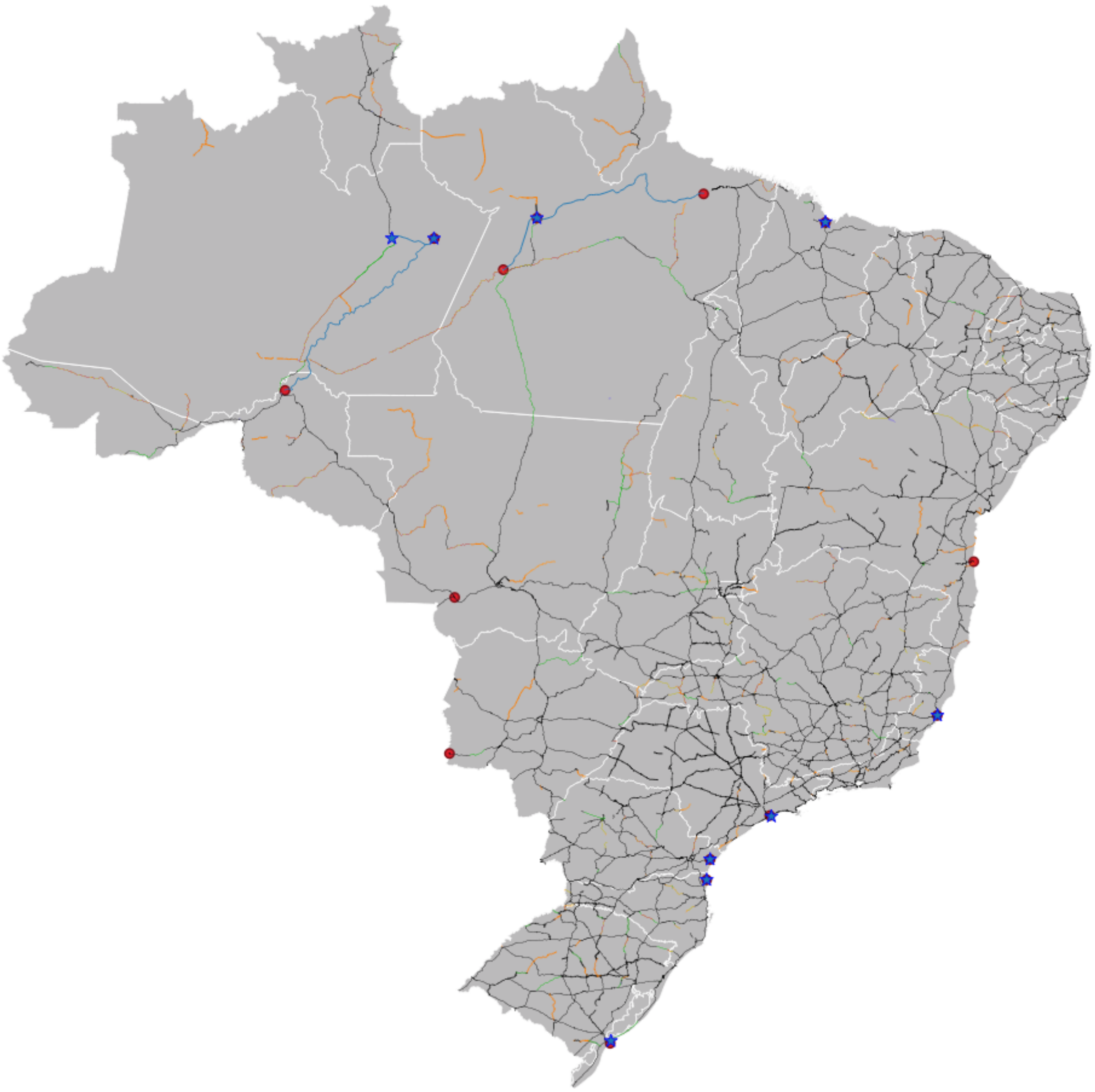

DNIT road network and soy export ports (blue stars) for year 2005. Red dots are internal soy ports, that transfer to export ports through waterways (blue lines). Paved roads in black. Dirt roads in orange.

Reconstructing 2000 road network

```
g.copy vect=dnit2005,dnit2000 --overwrite
v.db.dropcolumn map=dnit2000
columns=br,uf,codigo,local_ini,local_fim,km_inic,km_fina,extensao,superficie,tr_coincid,est_coincid,versao
db.in.ogr input=input/roads/PNV/PNV2000.csv output=pnv2000 --overwrite
v.db.join map=dnit2000 column=vl_codigo other_table=pnv2000 other_column=codigo
db.select sql='select superficie, count() from dnit2000 group by superficie'
```

superficie	count()

superficie	count()
	1356
DUP	504
EOD	87
EOI	6
EOP	125
IMP	396
LEN	108
PAV	3571
PLA	47
TRV	28

`./fix_road_attrib.py dnit2000 pnv2000 -r matches_2000.txt`

New status of the unclassified roads will be:

Status	count
PAV	890
EOD	13
EOI	8
LEN	36
IMP	156
None	58
EOP	37
PLA	39
DUP	111
TRV	8

After substitution, road status are:

superficie	count()
	58
DUP	615
EOD	100
EOI	14

superficie	count()
EOP	162
IMP	552
LEN	144
PAV	4461
PLA	86
TRV	36

```
v.extract -r input=dnit2000 where="superficie is 'PLA' or superficie is null" output=dnit2000c
g.rename --o vect=dnit2000c,dnit2000
```

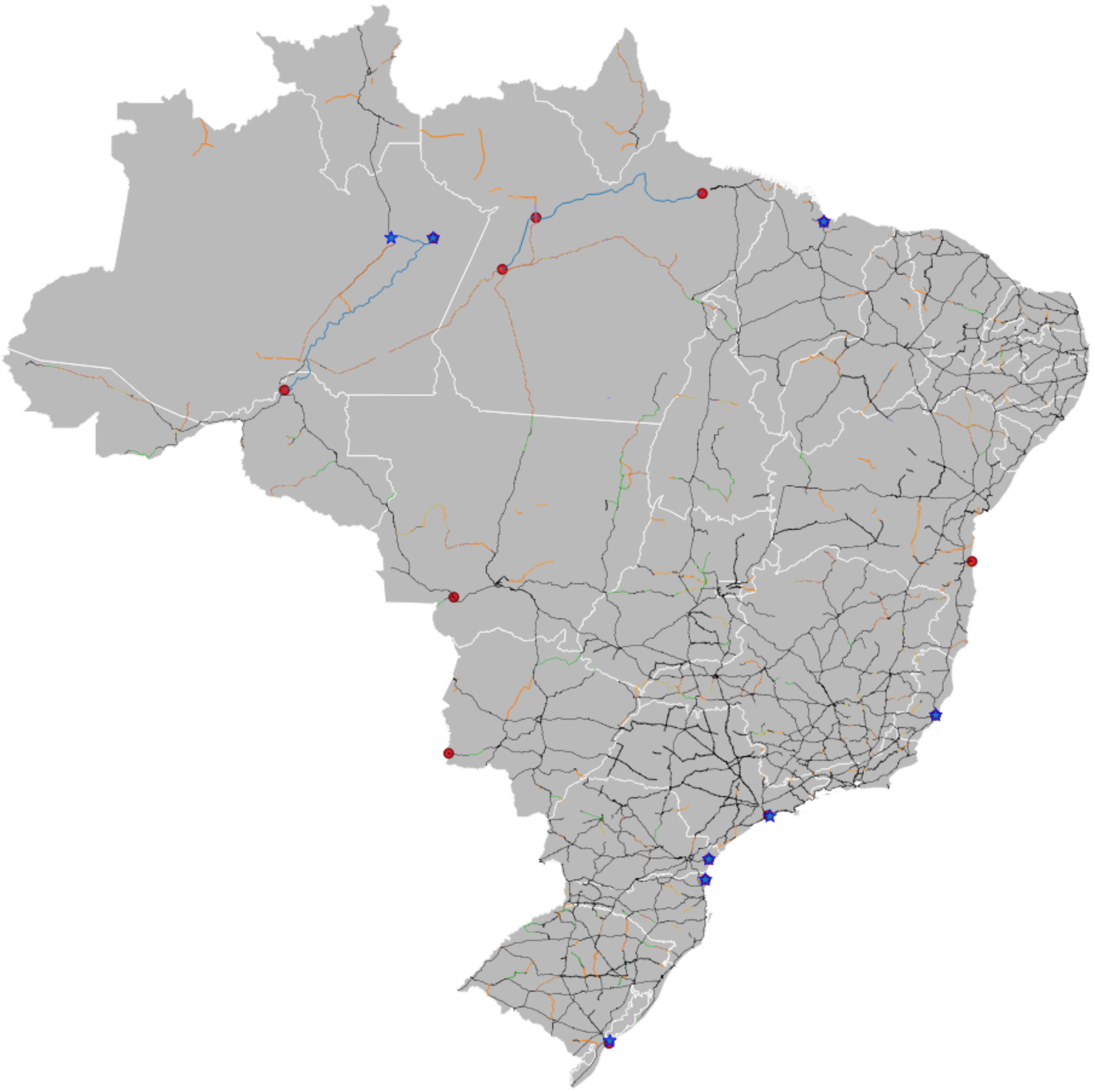

DNIT road network and soy export ports (blue stars) for year 2000. Red dots are internal soy ports, that transfer to export ports through waterways (blue lines). Paved roads in black. Dirt roads in orange.

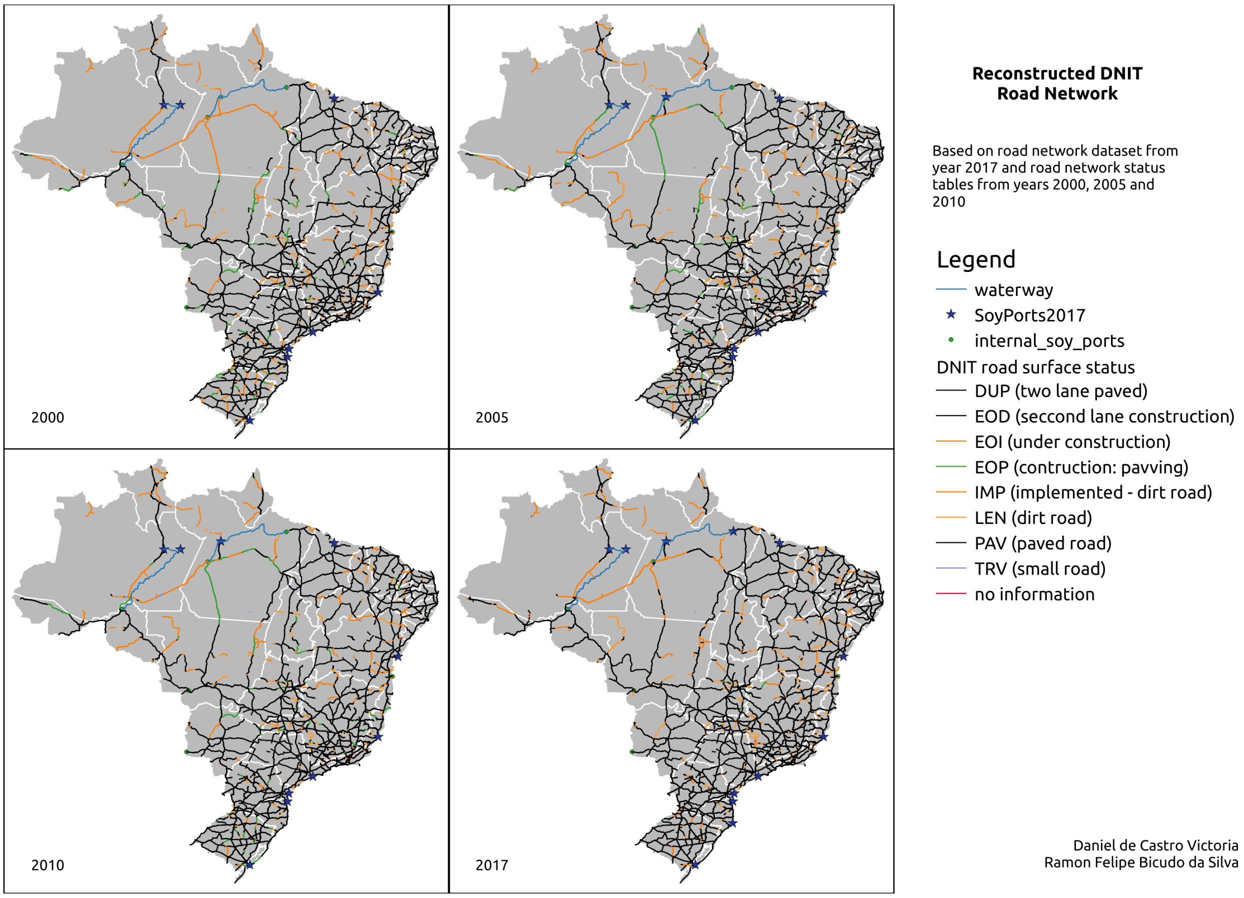

Comparison of the four road networks and road status.

Rail and waterways

r.cost algorithm treats rail and waterways as if they were roads. You could *board* anywhere. Thus, we are not considering rail and waterways in this analysis. The important part is to get an idea of the changes in the relative cost of transportation along the years.

Prepare data for cost analysis in GRASS

Cost analysis in GRASS can be very fast if done using raster format. No need to create a cleaned and connected network. On the down side, if the network is not connected, the cost of traversing a area with a gap will be higher.

Analysis is being done at 36 arcsec resolution.

`g.region -p`
projection: 3 (Latitude-Longitude)
zone: 0
datum: wgs84
ellipsoid: wgs84
north: 6N
south: 34S
west: 75W
east: 34W
nsres: 0:00:36
ewres: 0:00:36
rows: 4000
cols: 4100
cells: 16400000

Cost for each cell will be considered as follows:

Type	Cost
no network	50
Unpaved road	36
Paved road	16

Cost are set on relative terms, i.e. for unpaved road it is more than twice the cost of paved roads. DNIT surface status will be used for classifying paved / unpaved.

Cost analysis for 2017 road network

Add cost column to road vector map, convert to raster and remove cost column.

```
v.db.addcolumn map=dnit2017 columns='cost INT'
```

What are the surface road types?

```
db.select sql='select distinct vl_surface from dnit2017'
```

Surface type *TRV (travessia)* is a river crossing with no bridge. That's a high cost! So I'll not add a value and it will be treated as a *no network* cell.

```
v.db.update map=dnit2017 column=cost value=32 where="vl_surface IN ('LEN', 'EOI', 'IMP', 'EOP')"
v.db.update map=dnit2017 column=cost value=16 where="vl_surface IN ('PAV', 'EOD', 'DUP')"
v.to.rast input=dnit2017 output=dnit2017_rast use=attr attribute_column=cost
```

Calculate a cost for cells with no road/rail

```
echo rest = if(brasil, 50) | r.mapcalc
```

Join the maps

```
r.patch -z input=dnit2017_rast,rest output=road_cost2017
g.remove -f rast name=rest
```

Note to self: Don't forget to fix color table after r.patch

Prepare start_points map (2017 ports + Porto Velho)

```
v.extract input=internal_soy_ports output=porto_velho cats=12 --overwrite
v.patch input=SoyPorts2017,porto_velho output=allPorts_2017 --overwrite
g.remove -f vect name=porto_velho
```

Run cost analysis

```
r.cost -k input=road_cost2017 output=road_distance2017 start_points=allPorts_2017 max_cost=0 memory=300 --overwrite
```

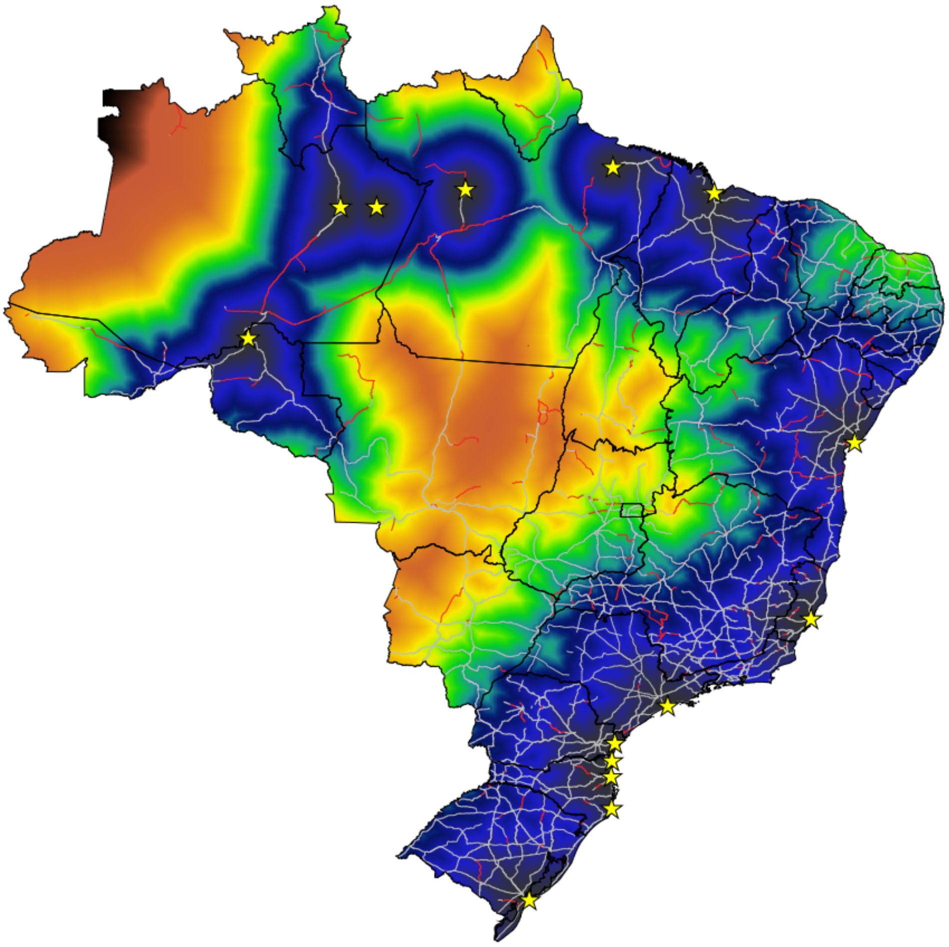

Road cost to port, year 2017. Ports shown as yellow stars. Roads in light gray (paved) or red (unpaved).

Cost analysis for 2010 road network

Add cost column to road vector map, convert to raster and remove cost column.

```
v.db.addcolumn map=dnit2010 columns='cost INT'
```

What are the surface road types?

```
db.select sql='select distinct vl_surface from dnit2010'
```

Surface type *TRV (travessia)* is a river crossing with no bridge. That's a high cost! So I'll not add a value and it will be treated as a *no network* cell.

```
v.db.update map=dnit2010 column=cost value=32 where="vl_surface IN ('LEN', 'EOI', 'IMP', 'EOP')"
v.db.update map=dnit2010 column=cost value=16 where="vl_surface IN ('PAV', 'EOD', 'DUP')"
v.to.rast input=dnit2010 output=dnit2010_rast use=attr attribute_column=cost
```

Calculate a cost for cells with no road/rail

```
echo rest = if(brasil, 50) | r.mapcalc
```

Join the maps

```
r.patch -z input=dnit2010_rast,rest output=road_cost2010
g.remove -f rast name=rest
```

Prepare start_points map (2010 ports + Porto Velho)

```
v.extract input=internal_soy_ports output=porto_velho cats=12 --overwrite
v.patch input=SoyPorts2010,porto_velho output=allPorts_2010 --overwrite
g.remove -f vect name=porto_velho
```

Run cost analysis

```
r.cost -k input=road_cost2010 output=road_distance2010 start_points=allPorts_2010 max_cost=0 memory=300 --overwrite
```

```
r.colors map=road_distance2010 rast=road_distance2017
```

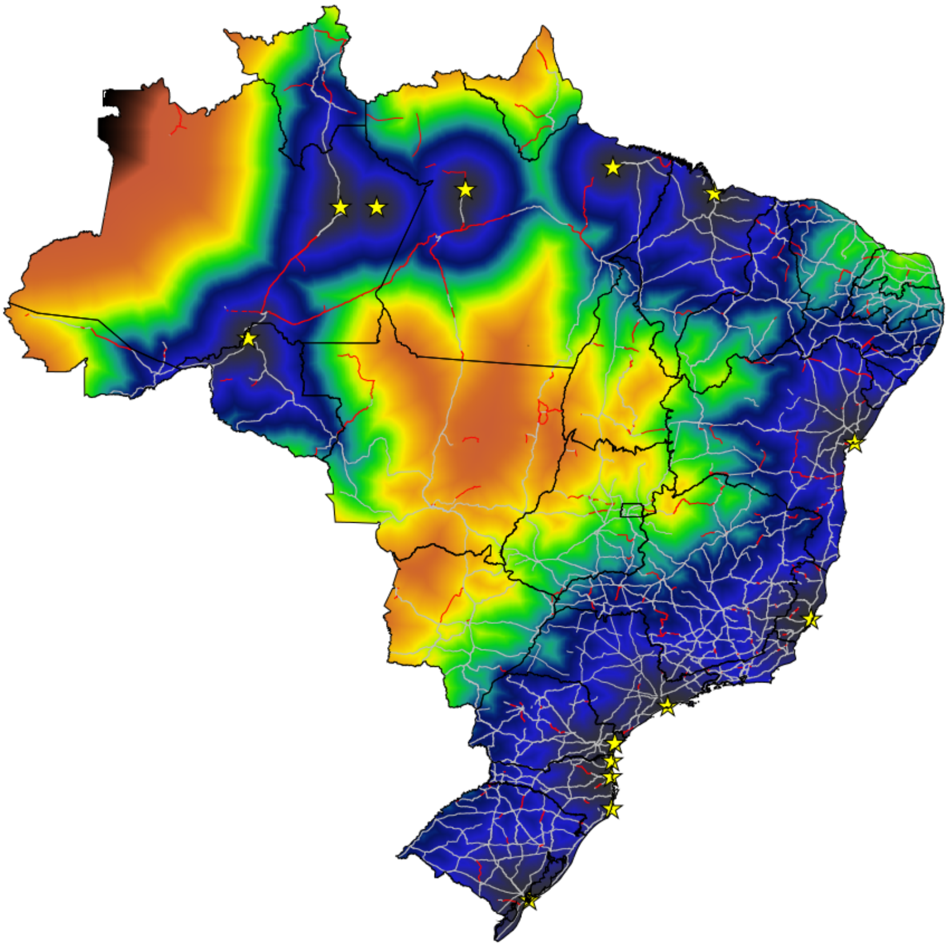

Road cost to port, year 2010. Ports shown as yellow stars. Roads in light gray (paved) or red (unpaved).

Cost analysis for 2005 road network

Add cost column to road vector map, convert to raster and remove cost column.

```
v.db.addcolumn map=dnit2005 columns='cost INT'
```

What are the surface road types?

```
db.select sql='select distinct vl_surface from dnit2005'
```

Surface type *TRV (travessia)* is a river crossing with no bridge. That's a high cost! So I'll not add a value and it will be treated as a *no network* cell.

```
v.db.update map=dnit2005 column=cost value=32 where="vl_surface IN ('LEN', 'EOI', 'IMP', 'EOP')"
v.db.update map=dnit2005 column=cost value=16 where="vl_surface IN ('PAV', 'EOD', 'DUP')"
v.to.rast input=dnit2005 output=dnit2005_rast use=attr attribute_column=cost
```

Calculate a cost for cells with no road/rail

```
echo "rest = if(brasil, 50)" | r.mapcalc
```

Join the maps

```
r.patch -z input=dnit2005_rast,rest output=road_cost2005
g.remove -f rast name=rest
```

Prepare start_points map (2005 ports + Porto Velho)

```
v.extract input=internal_soy_ports output=porto_velho cats=12 --overwrite
v.patch input=SoyPorts2005,porto_velho output=allPorts_2005 --overwrite
g.remove -f vect name=porto_velho
```

Run cost analysis

```
r.cost -k input=road_cost2005 output=road_distance2005 start_points=allPorts_2005 max_cost=0 memory=300 --
overwrite
r.colors map=road_distance2005 rast=road_distance2017
```

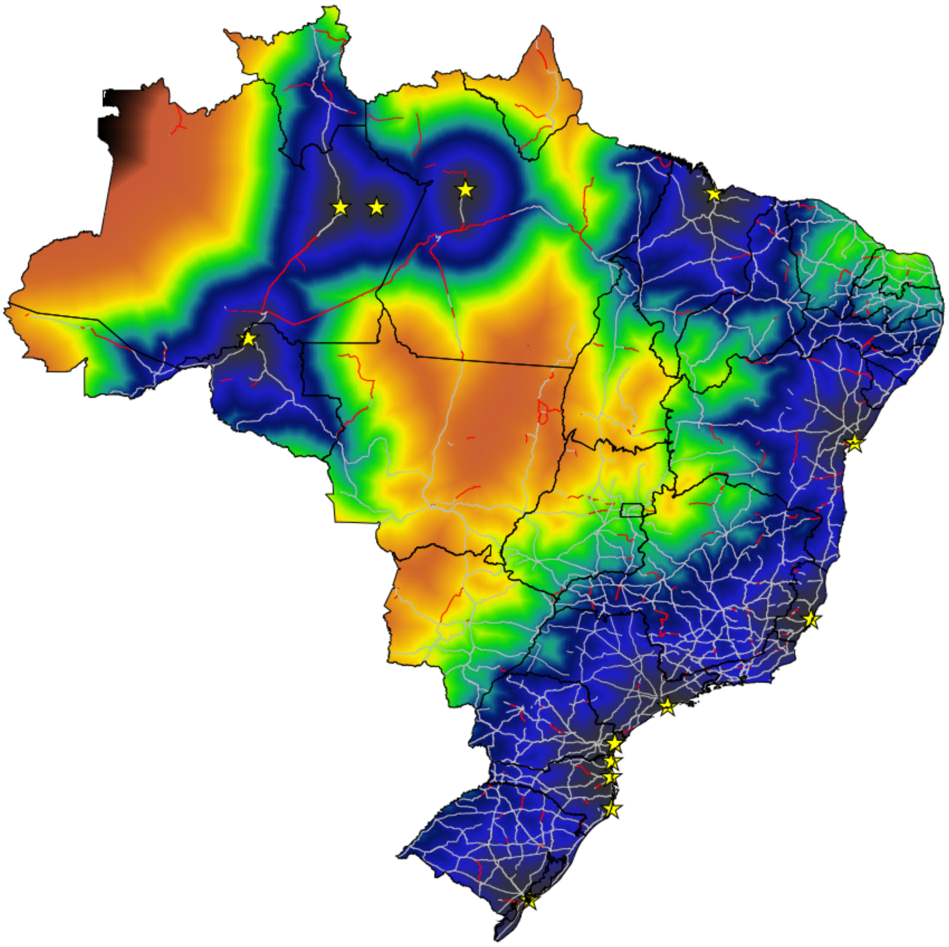

Road cost to port, year 2005. Ports shown as yellow stars. Roads in light gray (paved) or red (unpaved).

Cost analysis for 2000 road network

Add cost column to road vector map, convert to raster and remove cost column.

```
v.db.addcolumn map=dnit2000 columns='cost INT'
```

What are the surface road types?

```
db.select sql='select distinct vl_surface from dnit2000'
```

Surface type *TRV (travessia)* is a river crossing with no bridge. That's a high cost! So I'll not add a value and it will be treated as a *no network* cell.

```
v.db.update map=dnit2000 column=cost value=32 where="vl_surface IN ('LEN', 'EOI', 'IMP', 'EOP')"
v.db.update map=dnit2000 column=cost value=16 where="vl_surface IN ('PAV', 'EOD', 'DUP')"
v.to.rast input=dnit2000 output=dnit2000_rast use=attr attribute_column=cost
```

Calculate a cost for cells with no road/rail

```
echo "rest = if(brasil, 50)" | r.mapcalc
```

Join the maps

```
r.patch -z input=dnit2000_rast,rest output=road_cost2000
g.remove -f rast name=rest
```

Prepare start_points map (2000 ports + Porto Velho)

```
v.extract input=internal_soy_ports output=porto_velho cats=12 --overwrite
v.patch input=SoyPorts2000,porto_velho output=allPorts_2000 --overwrite
g.remove -f vect name=porto_velho
```

Run cost analysis

```
r.cost -k input=road_cost2000 output=road_distance2000 start_points=allPorts_2000 max_cost=0 memory=300 --overwrite
r.colors map=road_distance2000 rast=road_distance2017
```

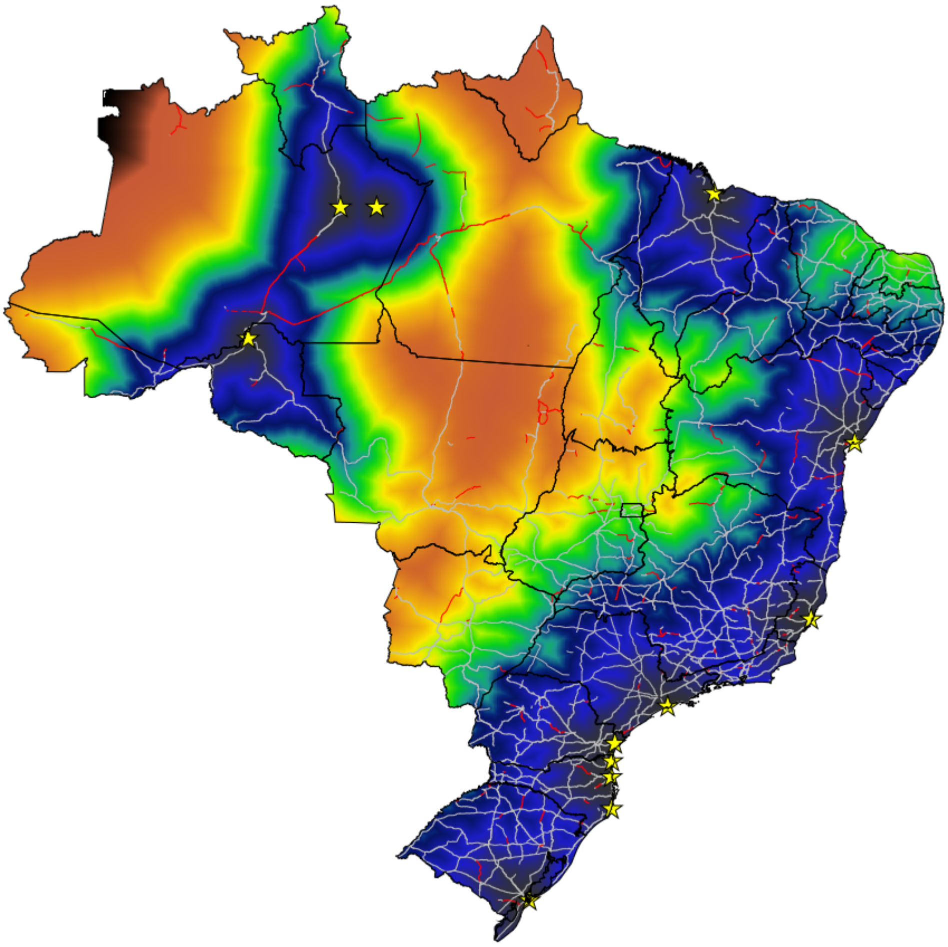

Road cost to port, year 2000. Ports shown as yellow stars. Roads in light gray (paved) or red (unpaved).

Simple quality check

Changes from year to year are not large and the same cost values for each surface type were used from year to year. So it's expected that the transport cost surface maps will not show large differences and will have similar values. This can be checked by looking at the Transport Cost surface maps side by side or by checking the raster statistical properties, obtained by using the `r.quantile` command.

Transport Cost quantiles for years 2000, 2005, 2010 and 2017

Cost map	25%	50%	75%

Cost map	25%	50%	75%
Road 2017	8547	13738	20506
Road 2010	8581	13780	20569
Road 2005	8941	14279	21090
Road 2000	9367	15179	22801

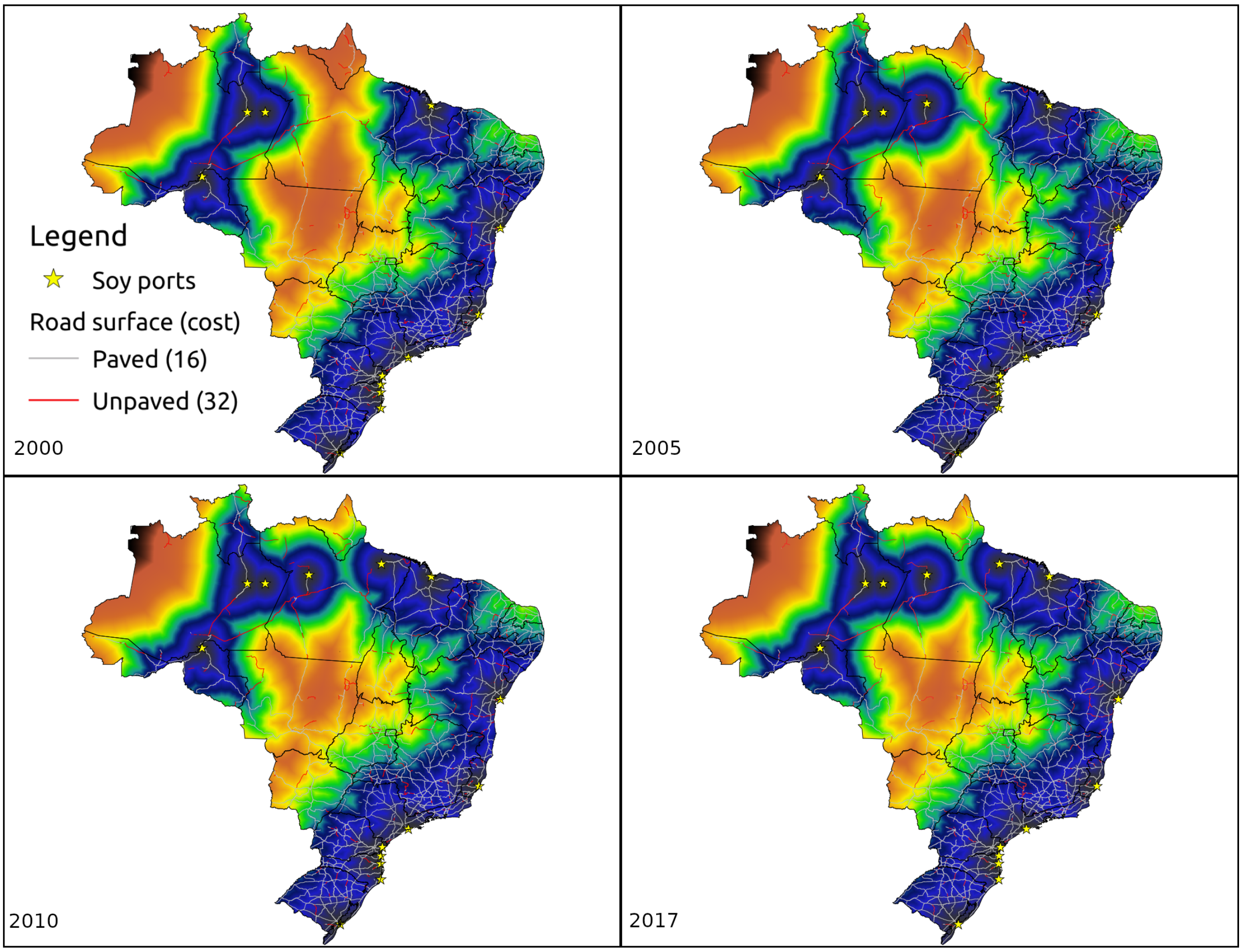

Cumulative cost distance to port, for years 2000, 2005, 2010 and 2017.
